# Causes of In-Hospital Death and Pharmaceutical Associations with Age of Death during a 10-Year Period (2011–2020) in Individuals with and without Diabetes at a Japanese Community General Hospital

**DOI:** 10.3390/jcm13051283

**Published:** 2024-02-24

**Authors:** Minae Hosoki, Taiki Hori, Yousuke Kaneko, Kensuke Mori, Saya Yasui, Seijiro Tsuji, Hiroki Yamagami, Saki Kawata, Tomoyo Hara, Shiho Masuda, Yukari Mitsui, Kiyoe Kurahashi, Takeshi Harada, Shingen Nakamura, Toshiki Otoda, Tomoyuki Yuasa, Akio Kuroda, Itsuro Endo, Munehide Matsuhisa, Ken-ichi Aihara

**Affiliations:** 1 Department of Internal Medicine, Anan Medical Center, 6-1 Kawahara, Takarada-cho, Tokushima 774-0045, Japan; minae.energy.flow@gmail.com (M.H.); taiki4725@yahoo.co.jp (T.H.); yousukekaneko115@gmail.com (Y.K.); m0r1.sword@gmail.com (K.M.); sayapon626@outlook.jp (S.Y.); tsuji.seiji89@gmail.com (S.T.); saki.0903.c5@gmail.com (S.K.); 2 Department of Internal Medicine, Tokushima Prefectural Kaifu Hospital, 266 Sugitani, Nakamura, Tokushima 775-0006, Japan; 3 Department of Hematology, Endocrinology and Metabolism, Tokushima University Graduate School of Biomedical Sciences, Tokushima 770-8503, Japan; yamagami.hiroki@tokushima-u.ac.jp (H.Y.); hara.tomoyo@tokushima-u.ac.jp (T.H.); t.k.shiho@gmail.com (S.M.); mitsui.yukari@tokushima-u.ac.jp (Y.M.); takeshi_harada@tokushima-u.ac.jp (T.H.); shingen@tokushima-u.ac.jp (S.N.); 4 Department of Diabetes and Metabolism, Tokushima Prefectural Central Hospital, 1-10-3 Kuramoto-cho, Tokushima 770-8539, Japan; 5 Department of Community Medicine for Respirology, Hematology and Metabolism, Tokushima University Graduate School of Biomedical Sciences, 3-18-15 Kuramoto-cho, Tokushima 770-8503, Japan; kurahashi.kiyoe@tokushima-u.ac.jp; 6 Department of Community Medicine and Medical Science, Tokushima University Graduate School of Biomedical Sciences, 3-18-15 Kuramoto-cho, Tokushima 770-8503, Japan; otoda.toshiki@tokushima-u.ac.jp (T.O.); yuasa.tomoyuki@tokushima-u.ac.jp (T.Y.); 7 Diabetes Therapeutics and Research Center, Institute of Advanced Medical Sciences, Tokushima University, 3-18-15 Kuramoto-cho, Tokushima 770-8503, Japan; kurodaakio@tokushima-u.ac.jp (A.K.); matuhisa@tokushima-u.ac.jp (M.M.); 8 Department of Bioregulatory Sciences, Tokushima University Graduate School of Biomedical Sciences, 3-18-15 Kuramoto-cho, Tokushima 770-8503, Japan; endoits@tokushima-u.ac.jp

**Keywords:** cause of death, pharmaceutical associations, age of death, 10-year period, diabetes

## Abstract

Since diabetes and its complications have been thought to exaggerate cardiorenal disease, resulting in a short lifespan, we investigated causes of death and lifespans in individuals with and without diabetes at a Japanese community general hospital during the period from 2011 to 2020. Causes of death and age of death in individuals with and those without diabetes were compared, and associations between medications used and age of death were statistically analyzed. A total of 2326 deaths were recorded during the 10-year period. There was no significant difference between the mean ages of death in individuals with and those without diabetes. Diabetic individuals had higher rates of hepato-pancreatic cancer and cardio-renal failure as causes of death. The prescription rates of antihypertensives, antiplatelets, and statins in diabetic individuals were larger than those in non-diabetic individuals. Furthermore, the use of sulfonyl urea or glinides and insulin was independently and inversely associated with the age of death. In conclusion, individuals with diabetes were treated with comprehensive pharmaceutical interventions and had life spans comparable to those of individuals without diabetes. This study’s discovery of an inverse relationship between the use of insulin secretagogues or insulin and the age of death suggests that the prevention of life-threatening hypoglycemia is crucial for individuals with diabetes.

## 1. Introduction

Diabetes, including type 1 diabetes (T1D) and type 2 diabetes (T2D), is associated with increased risks of cancers, cardiovascular disease (CVD), liver disease and death. According to a survey conducted from 2001 to 2010, the average ages of death of Japanese individuals with diabetes (males: 71.4 years, females: 75.1 years) were younger than the average life expectancies of Japanese in 2010 (males: 79.6 years, females: 86.3 years) [1]. On the other hand, Goto et al. estimated the exact causes of death, mortality rate and life expectancy of individuals with diabetes based on death records during the period from 1995 to 2001 in a diabetes specialist clinic in Japan [2]. Their study revealed that males and females with diabetes at the age of 40 years can expect to live for 39.2 years and 43.6 years, respectively, suggesting the life expectancy of individuals with diabetes is the same as that of non-diabetic individuals [2].

The life expectancy of patients with diabetes and patients with cardiovascular diseases has improved due not only to recent advances in treatment but also increased awareness of the importance of lifestyle improvements. In fact, recent studies have shown that a healthy lifestyle, such as aerobic exercise, a balanced diet, maintaining normal body mass index and not smoking, contributes to longevity in various populations [3,4,5].

Taken together, advances in comprehensive treatments, including lifestyle modifications, diet therapy and pharmaceutical interventions, for diabetes and its complications may have resulted in an increase in the lifespan of diabetic individuals. 

In fact, several clinical trials on multifactorial intervention, including the Steno-2 study [6,7], the Anglo–Danish–Dutch Study of Intensive Treatment In People with Screen-Detected Diabetes in Primary Care (ADDITION-Europe) [8], the Japan Diabetes Optimal Treatment study for 3 major risk factors of cardiovascular diseases (J-DOIT3) [9], the Nephropathy In Diabetes Type 2 (NID-2) study [10] and Diabetic Nephropathy Remission and Regression Team Trial in Japan (DNETT-Japan) study [11], have shown reductions in cardiovascular and renal events, though not necessarily significant [12]. Therefore, we need to know from epidemiological investigations whether diabetes and related medications influence the cause of death and lifespan in recent populations. 

Since Anan City (https://en.wikipedia.org/wiki/Anan,_Tokushima (accessed on 8 October 2023)), which is located in the southern area of Tokushima Prefecture, in the eastern part of Shikoku Island, Japan, has a population of approximately 70,000 and a population composition that is similar to the average population composition in Japan (Figure S1) and since Anan Medical Center is a flagship community general hospital in Anan City that has 398 beds and consists of 22 clinical departments, we think that analyses of the causes of in-hospital death and prescription drugs before death in individuals with and those without diabetes in our community hospital can be linked to an understanding of the causes of death and promotion of the extension of a healthy life expectancy in Japan. 

The purpose of this study was to investigate the age of death, causes of in-hospital death, comorbidities, and the association between medications taking during outpatient clinic visits before death in individuals with and those without diabetes, using electronic medical records during the period from 2011 to 2020 at our community general hospital.

## 2. Materials and Methods

### 2.1. Study Design, Subjects and Ethics Statement

A retrospective analysis of the causes of death (2336 deaths) in hospitalized patients at Anan Medical Center during the period from 2011 to 2020 was conducted. Information on clinical characteristics of the patients was obtained from electronic medical records in Anan Medical Center. The study endpoints were age of death and causes of in-hospital death (pneumonia; other infections; renal failure; neoplasms including lung cancer, esophageal cancer, colorectal cancer, liver cancer, pancreatic cancer, breast cancer, uterine cancer, leukemia, lymphoma, myelodysplastic syndromes (MDS) and other neoplasms; cerebrovascular diseases including cerebral infarction, cerebral hemorrhage and subarachnoid hemorrhage (SAH)*;* cardiovascular diseases including arrhythmia, myocardial infarction, and heart failure; insenescence; suicide; others; and unknown). Moreover, comorbidities (renal insufficiency, hemodialysis, cerebral infarction, cerebral hemorrhage, heart failure, atrial fibrillation, angina pectoris, myocardial infarction, hypertension, dyslipidemia, neoplasms, liver cirrhosis, depression and dementia), and medications provided by the outpatient clinic (angiotensin II receptor blockers (ARBs) or angiotensin-converting enzyme inhibitors (ACEis), calcium channel blockers (CCBs), β blockers, mineral corticoid receptor blockers (MR blockers), statins, antiplatelets, anticoagulants, diuretics, antacids, sleeping pills, sulfonyl urea (SU), dipeptidyl peptidase-4 (DPP-4) inhibitors, metformin, sodium glucose cotransporter 2 (SGLT2) inhibitors, α glucosidase inhibitors, pioglitazone, insulin and glucagon-like peptide-1 receptor agonists (GLP-1RAs)) were recorded. In addition to a description of diabetes diagnosis in the electronic medical records, individuals who had a value of hemoglobin A1c (HbA1c) ≥ 6.5% during outpatient clinic visits or on admission were defined as individuals with diabetes. Individuals with either T1D or T2D were included in this study. 

### 2.2. Statistical Analysis

Continuous variables were averaged and expressed as means ± standard deviation (SD). Categorical variables were compared by performing the χ^2^ test or Fisher’s exact test. For comparisons among two or three groups, we performed Student’s *t*-test or the Mann–Whitney U test and one-way ANOVA or Kruskal–Wallis’s test for numeric variables, depending on the variables’ distribution. Multivariate analysis was also carried out to determine the associations between medications and age of in-hospital death. The analyses were performed by using GraphPad Prism 10.1.0 (GraphPad Software, San Diego, CA, USA). The threshold for statistical significance was set at *p* < 0.05. 

## 3. Results

### 3.1. Ages of In-Hospital Death in Total Individuals, Individuals with Diabetes and Those without Diabetes

As shown in Table 1, a total of 2336 deaths, 48.4% of which were females, in the hospital during a 10-year period were analyzed. There were 570 deaths (including deaths in 326 males and 244 females) in individuals with diabetes. On the other hand, there were 1766 deaths (including deaths in 880 males and 886 females) in individuals without diabetes (Table 1). The overall ages of in-hospital death were 79.4 ± 10.4 years for males and 83.4 ± 10.6 years for females (Table 1). The ages of death in individuals with diabetes were 78.8 ± 8.9 years for males and 82.5 ± 9.7 years for females, and the ages of death in individuals without diabetes were 79.6 ± 10.9 years for males and 83.6 ± 10.9 years for females. There were no significant differences in the ages of death between individuals with diabetes and those without diabetes in both males and females (Table 1). A comparison of the ages of death during the first half of the decade (2011–2015) and during the second half of the decade (2016–2020) showed that the lifespan in the second half of the decade was significantly prolonged compared to that in the first half of the decade regardless of sex and diabetes (Table 1).

### 3.2. Causes of Death in Individuals with Diabetes and Those without Diabetes

**Figure 1:**
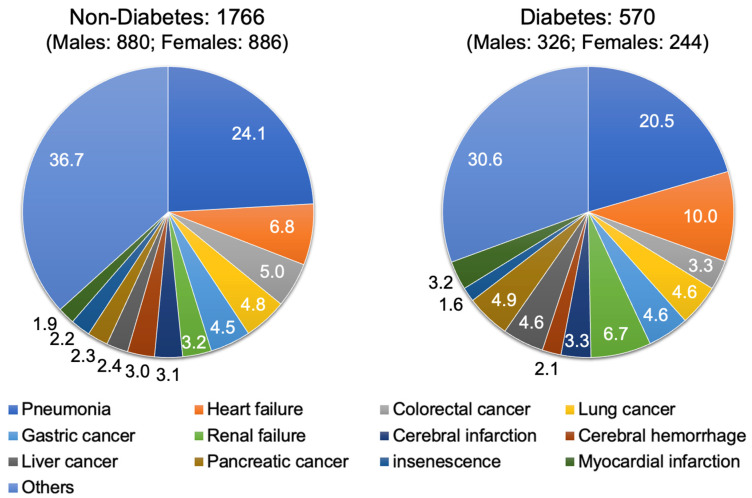
Pie charts of causes of in-hospital death in individuals with and those without diabetes at our community general hospital.

As shown in Table S1, the leading cause of death in the total number of individuals was pneumonia (23.2%) followed by heart failure (7.6%) and lung cancer (4.8%). In individuals without diabetes, the leading cause of death was pneumonia (24.1%) followed by heart failure (6.8%) and colorectal cancer (5.0%) (Figure 1). In individuals with diabetes, the leading cause of death was pneumonia (20.5%) followed by heart failure (10.0%) and renal failure (6.7%) (Figure 1).

**Figure 2:**
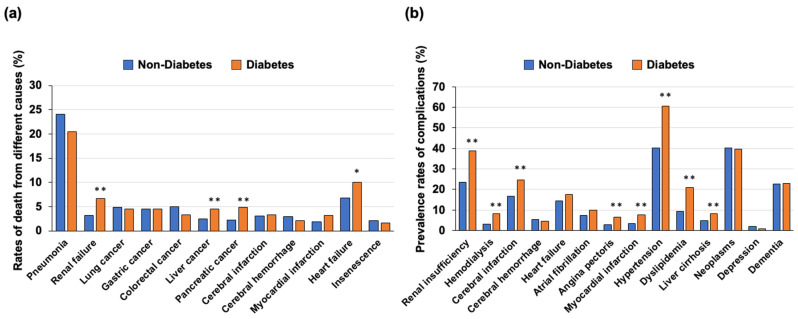
(**a**) Rates of death from different causes in individuals with and those without diabetes. (**b**) Rates of complications in individuals with and those without diabetes * *p* < 0.05, ** *p* < 0.01.

A comparison of the rates of death from different causes in individuals with and those without diabetes showed that the rates of death from renal failure, heart failure, liver cancer and pancreatic cancer were significantly higher in individuals with diabetes than in individuals without diabetes (Figure 2a). On the other hand, the rates of cause of death from pneumonia, cerebral infarction, cerebral hemorrhage, myocardial infarction, colorectal cancer, lung cancer, gastric cancer and insenescence were not significantly different between individuals with diabetes and those without diabetes as shown in Figure 2a. The results of a sub-analysis showed that the differences in the rates of death from different causes of death between individuals with and those without diabetes were very similar in males and females (Figure S2).

### 3.3. Complications in Individuals with Diabetes and Those without Diabetes

Regarding the complications in individuals with diabetes and those without diabetes, the prevalence rates of renal failure, hemodialysis, cerebral infarction, angina pectoris, myocardial infarction, hypertension, dyslipidemia and liver cirrhosis were significantly higher in individuals with diabetes than in individuals without diabetes. On the other hand, there were no significant differences in the prevalence rates of cerebral hemorrhage, heart failure, atrial fibrillation, malignancy, depression and dementia between the two groups (Figure 2b).

### 3.4. Medications Used in the Outpatient Clinic before Death

**Figure 3:**
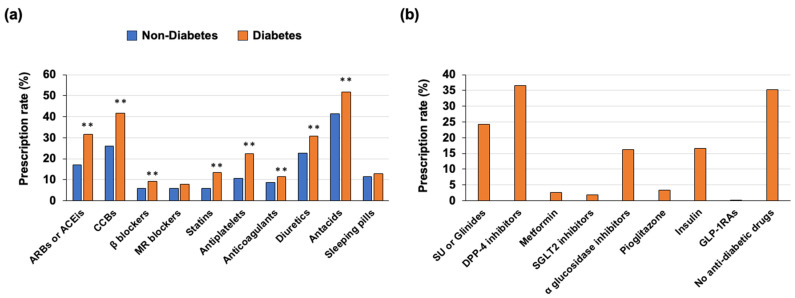
(**a**) Prescription rates of medications including hypotensive drugs, hypolipidemic drugs, antacids and sleeping pills before death in individuals with and those without diabetes. (**b**) Prescription rates of anti-diabetic drugs in individuals with diabetes ** *p* < 0.01.

We analyzed the medications used in the outpatient clinic before death in individuals with diabetes and those without diabetes (Figure 3a,b). The prescription rates of ARBs or ACEis, CCBs, β-blockers, statins, antiplatelets, anticoagulants, diuretics and antacids were significantly higher in the individuals with diabetes than in individuals without diabetes (Figure 3a). The prescription rates of MR blockers and sleeping pills were not significantly different between the individuals with diabetes and those without diabetes (Figure 3a).

In individuals with diabetes, the most frequently prescribed class of anti-diabetic drug was DPP-4 inhibitors, the second-most frequently prescribed drug was SU or glinides, and the third-most frequently prescribed drug was insulin (Figure 3b). Furthermore, relatively high rates of no anti-diabetic medications and low prescription rates of metformin, SGLT2 inhibitors and GLP-1RA were found (Figure 3b). The results for the prescription pattern of anti-diabetic drugs in this study may be linked to the idea that older people tend to decrease their food intake with gastrointestinal insufficiency and have impaired insulin secretion ability. Additionally, we found that insulin users had younger ages of death and higher mean HbA1c levels than those in individuals receiving anti-diabetic drugs other than insulin and individuals receiving no anti-diabetic drug (Table 2).

### 3.5. Identification of the Prescription Drugs Associated with Age of In-Hospital Death in Total Individuals, Individuals with Diabetes and Those without Diabetes 

To identify prescription drugs associated with age of in-hospital death, multivariate analysis including male gender, hypertension, dyslipidemia and diabetes as confounding factors was conducted in the total number of individuals. As shown in Table 3, male gender, dyslipidemia, SU or glinides, insulin and sleeping pills were independently and inversely associated with age of in-hospital death. Conversely, hypertension, antiplatelets, anticoagulants and diuretics were independently and positively associated with age of in-hospital death. Diabetes had no association with age of in-hospital death, even after adjustment of medications.

## 4. Discussion

An epidemiological analysis of 313,907 individuals with diabetes that was conducted between 2001 and 2018 in the UK showed that the total mortality rates decreased by 32% in males and 31% in females with diabetes [13]. With the decrease in vascular disease death rates, there has been a diversification of causes of death, including non-CVDs in individuals with diabetes, and cancer has become the primary cause of diabetes-related death instead of vascular diseases [13]. It was suggested in the report that comprehensive preventative interventions are reasons for this trend of a decrease in mortality risk in individuals with diabetes [13]. Likewise, as shown in our study, the lifespan of the individuals with diabetes was significantly prolonged in the second half of the decade compared to that in the first half of the decade. As shown in the study conducted in the UK [13], diversification in causes of death was also found in this study. 

Despite the fact that neoplasms are the most common cause of death in Japan, the number of deaths due to CVDs, including heart disease and cerebrovascular diseases, are almost the same as the number of deaths due to neoplasms. Thus, in addition to the prevention and treatment of neoplasms, the prevention and treatment of CVDs are very important issues for a healthy life expectancy.

The Hisayama study, a longitudinal epidemiological study conducted in Japan, has shown that obesity, diabetes and dyslipidemia have recently become greater contributors to the development of cerebrovascular disorders. Therefore, it is well recognized that in order to prevent CVDs, it is important to manage not only hypertension but also obesity, diabetes, dyslipidemia and chronic kidney disease (CKD). Thus, the lifespan of individuals with such cardiovascular risk factors has been extended due to the rigorous development and dissemination of clinical guidelines for the treatment of each condition [14,15,16,17,18] by practicing physicians in Japan.

Due to the alarming increases in the prevalence of type 2 diabetes, much attention has been given to the relationship between diabetes and cancer. The Cancer Prevention Study II (CPS-II), started in 1982, is an investigation into the mortality of about 1.2 million American individuals, and the study showed, after 26 years of follow-up, that both men and women with diabetes were at an increased risk of death from liver, pancreatic, colon, and breast cancer [19]. Therefore, our results showing a higher rate of hepato-pancreatic cancer as a cause of death in individuals with diabetes are reasonable. As for liver cancer, the results in this study are thought to be due to the fact that individuals with diabetes are particularly susceptible to steatohepatitis, a condition with a strong risk for the development of liver cirrhosis and liver cancer. Furthermore, pancreatic cancer itself is a risk for the development of diabetes, and the relationship between the high prevalence of pancreatic cancer and presence of diabetes in this study is thought to include both a causal relationship and a reverse causal relationship.

In our study, since many complications, including cardiovascular and cerebrovascular diseases, renal diseases, hypertension, dyslipidemia and liver cirrhosis, were more frequently found in individuals with diabetes than in individuals without diabetes, prescription rates of anti-hypertensive agents, statins and antiplatelets were higher in individuals with diabetes than in individuals without diabetes. We believe that the results obtained for prescription drugs in this study indicate that clinicians in both community clinics and hospitals in Anan City Tokushima Japan have provided effective and comprehensive pharmaceutical interventions for individuals with diabetes. 

In this study, we showed that individuals with diabetes had life spans comparable to those of individuals without diabetes. We previously examined vascular endothelial function through flow-mediated dilation (FMD) in patients taking medication for hypertension, dyslipidemia, diabetes, and other lifestyle-related diseases, and we found that FMD values did not differ between individuals with and those without diabetes [20,21]. Therefore, maintaining vascular endothelial function through comprehensive pharmaceutical interventions may be one of the reasons why lifespans are not shortened due to diabetes.

Although the multiple linear regression analysis showed that hypertension and use of antiplatelets, anticoagulants and diuretics were positive contributors to an older age of in-hospital death, we speculate that the paradoxical relationship between these factors and lifespan is based on medication with anti-hypertensive agents that leads to vascular protection in individuals diagnosed with hypertension and a reverse causal relationship due to the increasing need for cardiovascular medications in much older people.

As for anti-diabetic drugs, the results of previous studies on the beneficial effects of metformin in the treatment of individuals with T2D suggest that metformin may also have therapeutic utility as an anti-aging drug that may also extend lifespan. In detail, metformin is likely to have positive effects on aging and lifespan [22,23,24,25,26] because it has anti-hyperglycemic effects, improves insulin sensitivity, reduces oxidative stress and protects the endothelium and vascular function [24,27]. On the other hand, this study did not show any clinical benefits of using metformin for the extension of lifespan in individuals with diabetes. We speculate that the low prescription rate of metformin in elderly individuals with diabetes is one of the reasons for the drug’s inability to extend lifespan in individuals with diabetes in this study.

The results of multiple regression analysis in this study showed that use of insulin secretagogues including SU and glinides was a negative contributor for age of death. This result is consistent with the results of a previous study showing that the use of glimepiride and standard SU doses led to an increase in severe hypoglycemic events in long-term nursing home residents in the United States [28]. 

The results of our multiple regression analysis also showed that use of insulin was a negative contributor for age of death. The Action to Control Cardiovascular Risk in Diabetes (ACCORD) study, in which mortality and macrovascular events were compared in a standard treatment group and an intensive treatment group in accordance with target HbA1c levels, showed that hypoglycemic events and mortality were increased in the intensive treatment group [29] and that symptomatic, severe hypoglycemia was associated with an increased risk of death in both the standard treatment group and intensive treatment group [30]. Although the hypothesis that insulin dose contributed to CV mortality was skeptically received [31,32], nocturnal hypoglycemia is very common and largely underdiagnosed in elderly individuals receiving insulin treatment T2D [33]. Taken together, the results indicate that adverse effects induced by insulin use may shorten the lifespan in individuals with diabetes. Furthermore, in this study, since the mean HbA1c level in insulin users was higher than the levels in both the group treated with anti-diabetic drugs other than insulin and the group not treated with anti-diabetic drugs, there is a possibility that elevated HbA1c levels in insulin users inversely influences the lifespan of individuals with diabetes.

## 5. Limitations

This study has several limitations. First, the deaths in this study were only deaths at Anan Medical Center and did not include deaths at home or in other medical institutions, and that might have led to bias in analyzing the causes of death and ages of death. Second, in our hospital, individuals with diabetes were treated by diabetologists, and it is therefore possible that many of the cases of diabetes treated at Anan Medical Center were cases with multiple comorbidities and cases that were difficult to control against hyperglycemia. Third, although we investigated the causes of death and comorbidities by referring to medical records retrospectively, it is difficult to standardize diagnostic criteria of the diseases, comorbidities and causes of death. Fourth, the lack of consideration of the treatment duration of each prescription drug in outpatient clinics makes it difficult to accurately assess the influence of pharmaceuticals on the age of death. Fifth, although cigarette smoking is a crucial factor for the decrement of longevity due to respiratory and cardiovascular complications, data for smoking status were lacking in this study. Sixth, due to missing values and data for diabetic microangiopathy, including retinopathy and neuropathy, those complications were not considered in the analysis. Seventh, the associations between the identified medications used and the age of death in this study evaluated by multiple regression analysis did not show causal relationships. Finally, the cross-sectional study was conducted at a single center with a relatively small sample size, and prospective studies are therefore needed to clarify the causes of death, morbidities, and how pharmaceuticals affect the age of death in individuals with and those without diabetes in the general community.

## 6. Conclusions

Our community clinicians provided comprehensive pharmaceutical interventions, including antihypertensives, antiplatelets agents and statins for the treatment of individuals with diabetes that led to life spans of individuals with diabetes being comparable to those of individuals without diabetes during a 10-year period from 2011 to 2020 in both males and females. The inverse relationship between the use of an insulin secretagogue or insulin and age of death firstly found in this study suggests that the careful monitoring and prevention of life-threatening hypoglycemia are crucial for extending healthy life expectancy in individuals with diabetes. 

As indicated in the limitations, due to the retrospective nature of this study, a larger prospective study is necessary to investigate ways to improve the prognosis of elderly individuals with or without diabetes, including a healthier lifespan.

## Figures and Tables

**Table 1:** Comparison of ages of in-hospital death at our community general hospital in the total number of individuals and individuals with and without diabetes.

	Total (2336)	Non-Diabetes (1766)	Diabetes (570)	Non-Diabetes vs. Diabetes
Period	2011–2020			
Males (age of death, years (*n*))	79.4 ± 10.4 (1206)	79.6 ± 10.9 (880)	78.8 ± 8.9 (326)	ns
Females (age of death, years (*n*))	83.4 ± 10.6 (1130)	83.6 ± 10.9 (886)	82.5 ± 9.7 (244)	ns
Period	2011–2015			
Males (age of death, years (*n*))	78.3 ± 10.6 (585)	78.6 ± 11.1 (429)	77.6 ± 8.9 (156)	ns
Females (age of death, years (*n*))	82.0 ± 11.0 (532)	82.3 ± 11.2 (418)	80.8 ± 10.4 (114)	ns
Period	2016–2020			
Males (age of death, years (*n*))	80.4 ± 10.1 (621) *	80.6 ± 10.5 (451) *	80.0 ± 8.8 (170) *	ns
Females (age of death, years (*n*))	84.6 ± 10.1 (598) **	84.8 ± 10.4 (468) **	83.9 ± 8.7 (130) **	ns

The values are presented as means  ±  SD, ns: not significant * *p* < 0.05 vs. period from 2011 to 2015 in each sex, ** *p* < 0.01 vs. period from 2011 to 2015 in each sex.

**Table 2:** Comparisons of ages of in-hospital death and HbA1c levels among the insulin user group, anti-diabetic drugs other than the insulin group and no anti-diabetic drug group.

	Insulin User Group (95)		Anti-Diabetic Drugs Other than Insulin Group (273)		No Anti-Diabetic Drug Group (202)	
	Males (56)	Females (39)	Males (156)	Females (117)	Males (114)	Females (88)
Age of death (years)	75.1 ± 10.3	76.6 ± 12.4	79.2 ± 8.0 *	83.3 ± 8.7 *	80.1 ± 9.1 **	84.0 ± 8.6 **
HbA1c (%) (sample number)	7.05 ± 1.73 (47)	7.63 ± 2.02 (35)	6.77 ± 1.32 (137)	6.75 ± 1.44 ** (103)	6.29 ± 0.96 *# (88)	6.51 ± 1.12 ** (69)

The values are presented as means  ±  SD. * *p* < 0.05 vs. insulin user group in each sex, ** *p* < 0.01 vs. insulin user group in each sex. # *p* < 0.05 vs. anti-diabetic drugs other than insulin group in males.

**Table 3:** Multiple regression analysis including medications during treatment at the outpatient clinic for age of death at our community general hospital.

Variables	*t* Value	VIF	*p* Value
Male	−8.961	1.031	<0.001
Hypertension	5.990	1.965	<0.001
Dyslipidemia	−1.967	2.132	0.049
Diabetes	−0.448	1.967	0.655
ARBs or ACEis	0.465	1.359	0.642
CCBs	0.505	1.691	0.614
β blockers	0.564	1.107	0.573
MR blockers	−1.158	1.166	0.247
Statins	−1.570	2.036	0.117
Ezetimibe	−0.095	1.076	0.924
Antiplatelets	3.592	1.144	0.001
Anticoagulants	2.720	1.097	0.007
Diuretics	2.461	1.261	0.014
Antacids	−0.396	1.089	0.693
Sleeping pills	−2.338	1.037	0.020
SU or Glinides	−2.721	1.363	0.007
DPP-4 inhibitors	0.022	1.555	0.983
Metformin	−0.296	1.055	0.768
SGLT2 inhibitors	−0.836	1.039	0.403
α glucosidase inhibitors	0.029	1.259	0.977
Pioglitazone	0.949	1.091	0.343
Insulin	−4.485	1.185	<0.001
GLP-1RAs	0.339	1.013	0.735

Abbreviations: ARBs: angiotensin II receptor blockers; ACEis: angiotensin-converting enzyme inhibitors; CCBs: calcium channel blockers; MR: mineral corticoid receptor; SU: sulfonyl urea; DPP-4: dipeptidyl peptidase-4; SGLT2: sodium glucose cotransporter 2; GLP-1RAs: glucagon-like peptide-1 receptor agonists; VIF: variance inflation factor.

## Data Availability

Data will be made available on reasonable request.

## Supplementary Materials

The following supporting information can be downloaded at https://www.mdpi.com/article/10.3390/jcm13051283/s1, Figure S1: Population compositions of Anan city and in all of Japan; Figure S2: (a) Rates of death from different causes in males with and those without diabetes, (b) Rates of death from different causes in females with and those without diabetes; Table S1: Causes of in-hospital death during the period from 2011 to 2020 at our community general hospital.
