# Supplementary material for: Causes of In-Hospital Death and Pharmaceutical Associations with Age of Death during a 10-Year Period (2011–2020) in Individuals with and without Diabetes at a Japanese Community General Hospital
Source: J Clin Med. 2024 Feb 24;13(5):1283. doi: 10.3390/jcm13051283 (PMC10932181; doi:10.3390/jcm13051283)
Supplement: Supplementary file 1 [file jcm-13-01283-s001.zip › jcm-2877617-supplementary.pdf]

Figure S1

Population compositions of Anan City and All Japan

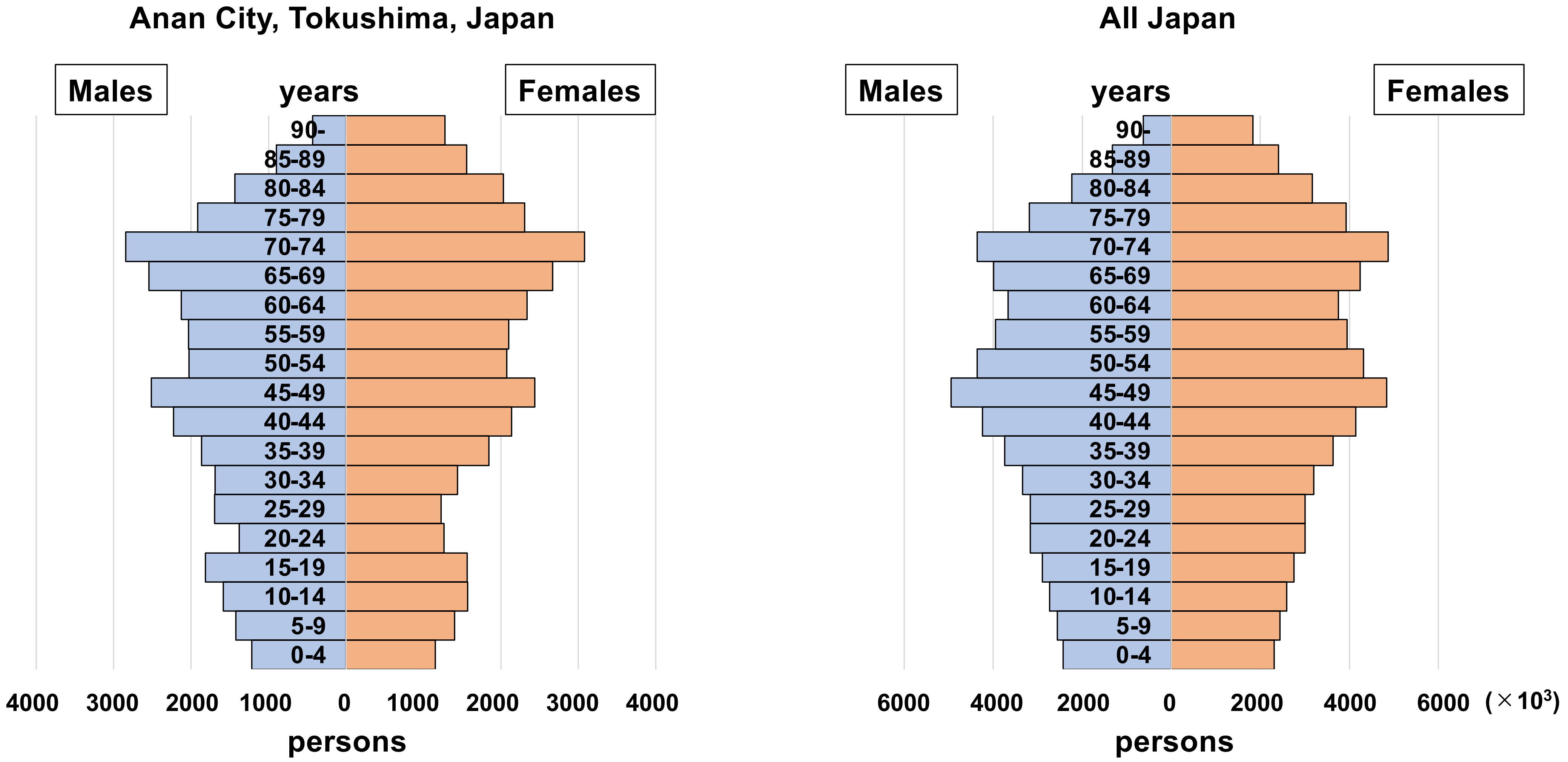

https://www.city.anan.tokushima.jp/docs/2021031900082/file_contents/setai.pdf

https://www.ipss.go.jp/site-ad/TopPageData/2020.png

Figure S2

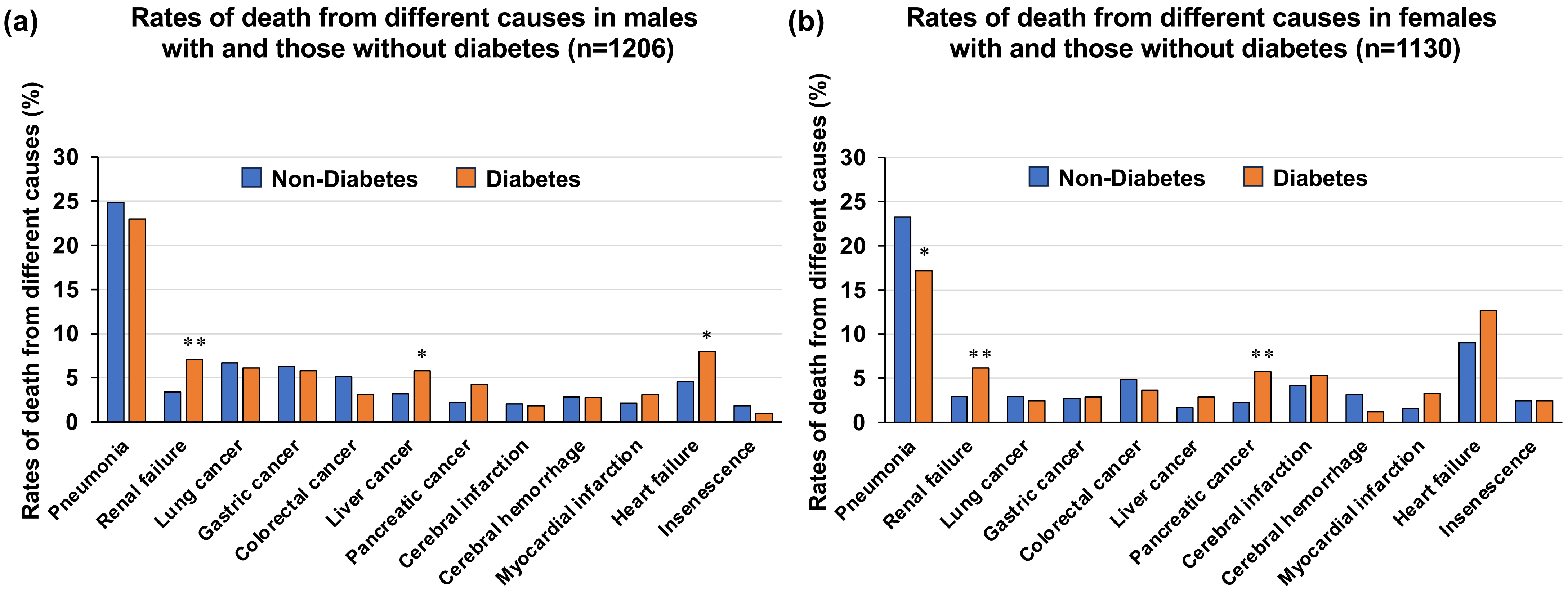

* $p < 0.05$, ** $p < 0.01$ vs non-diabetes

Table S1

Causes of in-hospital death during the period from 2011 to 2020 at our community general hospital

	All subjects (Total: 2336; Males: 1206; Females: 1130)						Non-Diabetes (Total: 1766; Males: 880; Females: 886)						Diabetes (Total: 570; Males: 326; Females: 244) T1D (7), T2D (498), Others or Unknown (65)					
	Males		Females		Total		Males		Females		Total		Males		Females		Total	
Cause of death	n	%	n	%	n	%	n	%	n	%	n	%	n	%	n	%	n	%
Pneumonia	294	24.4	248	21.9	542	23.2	219	24.9	206	23.3	425	24.1	75	23.0	42	17.2	117	20.5
Other infections	66	5.5	84	7.4	150	6.4	48	5.5	61	6.9	109	6.2	18	5.5	23	9.4	41	7.2
Renal failure**	53	4.4	41	3.6	94	4.0	30	3.4	26	2.9	56	3.2	23	7.1	15	6.1	38	6.7
Neoplasms	429	35.6	312	27.5	741	31.8	325	37.0	248	28.1	573	32.4	104	31.8	64	26.2	168	29.6
Lung cancer	79	6.6	32	2.8	111	4.8	59	6.7	26	2.9	85	4.8	20	6.1	6	2.5	26	4.6
Esophageal cancer	17	1.4	3	0.3	20	0.9	17	1.9	2	0.2	19	1.1	0	0.0	1	0.4	1	0.2
Gastric cancer	74	6.1	31	2.7	105	4.5	55	6.3	24	2.7	79	4.5	19	5.8	7	2.9	26	4.6
Colorectal cancer	55	4.6	52	4.6	107	4.6	45	5.1	43	4.9	88	5.0	10	3.1	9	3.7	19	3.3
Liver cancer**	47	3.9	22	1.9	69	3.0	28	3.2	15	1.7	43	2.4	19	5.8	7	2.9	26	4.6
Pancreatic cancer**	34	2.8	34	3.0	68	2.9	20	2.3	20	2.3	40	2.3	14	4.3	14	5.7	28	4.9
Breast cancer	0	0.0	14	1.2	14	0.6	0	0.0	13	1.5	13	0.7	0	0.0	1	0.4	1	0.2
Uterine cancer	0	0.0	10	0.9	10	0.4	0	0.0	9	1.0	9	0.5	0	0.0	1	0.4	1	0.2
Leukemia	12	1.0	9	0.8	21	0.9	10	1.1	8	0.9	18	1.0	2	0.6	1	0.4	3	0.5
Lymphoma	15	1.2	15	1.3	30	1.3	13	1.5	12	1.4	25	1.4	2	0.6	3	1.2	5	0.9
MDS	1	0.1	4	0.4	5	0.2	0	0.0	4	0.5	4	0.2	1	0.3	0	0.0	1	0.2
Other neoplasms	95	7.9	86	7.6	181	7.7	78	8.9	72	8.1	150	8.5	17	5.2	14	5.7	31	5.4
Cerebrovascular Diseases	60	5.0	96	8.4	156	6.7	45	5.0	79	9.0	124	7.0	15	4.6	17	6.9	32	5.6
Cerebral infarction	24	2.0	50	4.4	74	3.2	18	2.0	37	4.2	55	3.1	6	1.8	13	5.3	19	3.3
Cerebral hemorrhage	34	2.8	31	2.7	65	2.8	25	2.8	28	3.2	53	3.0	9	2.8	3	1.2	12	2.1
SAH	2	0.2	15	1.3	17	0.7	2	0.2	14	1.6	16	0.9	0	0.0	1	0.4	1	0.2
Cardiovascular Diseases	103	8.6	136	12.0	239	10.3	65	7.4	97	10.9	162	9.2	38	11.7	39	16.0	77	13.6
Arrhythmia	8	0.7	3	0.3	11	0.5	6	0.7	3	0.3	9	0.5	2	0.6	0	0.0	2	0.4
Myocardial infarction	29	2.4	22	1.9	51	2.2	19	2.2	14	1.6	33	1.9	10	3.1	8	3.3	18	3.2
Heart failure*	66	5.5	111	9.8	177	7.6	40	4.5	80	9.0	120	6.8	26	8.0	31	12.7	57	10.0
Insenescence	19	1.6	28	2.5	47	2.0	16	1.8	22	2.5	38	2.2	3	0.9	6	2.5	9	1.6
Suicide	2	0.2	3	0.3	5	0.2	2	0.2	3	0.3	5	0.3	0	0.0	0	0.0	0	0.0
Others	167	13.8	170	15.0	337	14.4	120	13.6	136	15.3	256	14.5	47	14.4	34	13.9	81	14.2
Unknown	13	1.1	12	1.1	25	1.1	10	1.1	8	0.9	18	1.0	3	0.9	4	1.6	7	1.2
Total	1,206	100.0	1,130	100.0	2,336	100.0	880	100.0	886	100.0	1,766	100.0	326	100.0	244	100.0	570	100.0

MDS: myelodysplastic syndromes; SAH: subarachnoid hemorrhage   *p<0.05: Non-Diabetes vs Diabetes; **p<0.01: Non-Diabetes vs Diabetes
